# The interplay of poorly soluble drugs in dissolution from amorphous solid dispersions

**DOI:** 10.1016/j.ijpx.2024.100243

**Published:** 2024-03-30

**Authors:** Marcel Kokott, Jörg Breitkreutz, Raphael Wiedey

**Affiliations:** Institute of Pharmaceutics and Biopharmaceutics, Heinrich Heine University, Duesseldorf Universitaetsstr. 1, Duesseldorf 40225, Germany

**Keywords:** Fixed dose combination, Amorphous solid dispersion, Ritonavir, Lopinavir, Biorelevant dissolution, Poorly water soluble drugs

## Abstract

In recent years, the application of fixed dose combinations of antiretroviral drugs in HIV therapy has been established. Despite numerous therapeutic benefits, this approach poses several challenges for the formulation development especially when poorly soluble drugs are considered. Amorphous solid dispersions (ASD) thereby have gained considerable interest in the pharmaceutical field, however, mainly including binary systems containing only one drug and a polymer. The *co*-formulation of two amorphous drugs can be accompanied by an immense increase in the complexity of the system as exemplarily reported for ritonavir and lopinavir embedded in a composite polymer matrix of PVPVA. The present study aims to present a new formulation approach to overcome the well-documented interaction during dissolution. Two different polymers, PVPVA and HPMCAS were used to produce ASDs for both drugs individually via hot-melt extrusion. The embedding of lopinavir in the slower dissolving polymer HPMCAS, while using PVPVA for ritonavir was found to significantly improve the overall dissolution performance compared to the individual use of PVPVA as well as to the commercial product Kaletra®. In addition, the use of different grades of HPMCAS demonstrated the possibility to further modify the dissolution profile. For a preliminary biorelevant assessment, the selected formulations were tested in a biphasic dissolution setup.

## Highlights

• Development of a new combination approach for lopinavir and ritonavir.
• Separating the dissolution-onsets was key for the improvement of the overall dissolution performance.
• Embedding lopinavir in different grades of HPMCAS could be used for fine tuning of lopinavir and ritonavir supersaturation.
• Newly developed combination approach also showed superiority in the biphasic dissolution setup.

## Graphical abstract

**Figure:**
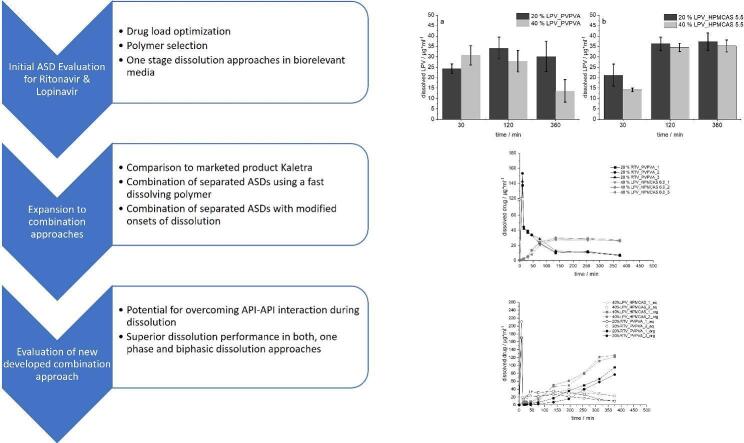

## 1 Introduction

In today's pharmaceutical development, the poor aqueous solubility of active pharmaceutical ingredients (API) is one of the major challenges (Baghel et al., 2016). The development of suitable formulations ensuring sufficient dissolution profiles is thereby of great importance, since a poor drug release and dissolution in the aqueous environment of the small intestine may result in a low bioavailability (BA) after oral drug administration (Miller et al., 2012). Of increased relevance in recent years, have been systems showing the ability to generate supersaturation of molecularly dissolved API within the intestinal fluids, such as amorphous solid dispersions (ASD) (Bevernage et al., 2013). The appropriate polymer selection is a key attribute to achieve a successfully performing ASD. Polymers are not only essential regarding the kinetic or thermodynamically driven prevention of API recrystallization in the solid state, but also play a major role in the drug release kinetics and the stabilization of supersaturation (Alonzo et al., 2010; He and Ho, 2015). An elaborate review by Schittny et al. summarized the physico-chemical properties of the polymer, the API-polymer interaction, and the respective homogeneity of the API in the polymer as most relevant aspects for the dissolution performance (Schittny et al., 2020). In terms of the influence on permeation, Borbas et al. highlighted the extent of supersaturation as a prerequisite for an increased flux across the membrane (Borbás et al., 2016). A linear correlation was found between the increase in flux and the extent of supersaturation up to a defined threshold, the “amorphous solubility”. Above this threshold no further increase in permeation was observed owing to the formation of a drug-rich colloidal phase, while the free drug concentration remained unchanged (Raina et al., 2014).

Substantial efforts have been made to study the complex dissolution and absorption behavior of ASDs, focusing generally on a single amorphous API dispersed in a polymer matrix (Kanzer et al., 2010; Ueda et al., 2019; Yang et al., 2023). The simultaneous application of two or more amorphous drugs, is much less reflected. The term co-amorphous has become widely known in the field, but usually applied to describe systems in which one low molecular weight compound (e.g. an amino acid) is used to stabilize an amorphous API (Dengale et al., 2016; Löbmann et al., 2013). In this context, however, the incorporation of two APIs into one polymer matrix is point of interest. Such combinations have gained considerable importance especially in HIV therapy, where co-formulated ritonavir (RTV) and lopinavir (LPV) are recommended as first line options (Welch et al., 2009). So called fixed-dose combination (FDC) provide relevance from the pharmacokinetic perspective since one of the compounds is often used to increase the effectiveness of the other API. This could be realized, for example, through the blockade of metabolizing enzymes, as it is present in the marketed product Kaletra®(Eichbaum et al., 2013). Additionally, the use of FDCs can reduce the daily number of tablets or capsules needed to be administered and finally improve patient compliance. Besides the highlighted therapeutic benefits, the incorporation of two amorphous APIs into one matrix can also go along with an immense increase in system complexity. A relevant pharmacokinetic study by Best et al. highlighted the immense loss in bioavailability for both APIs (embedded in PVPVA) when the film-coated tablet Kaletra® was altered prior to administration. They observed a significantly reduced LPV and RTV systemic exposure. This was expressed by a decrease in AUC for both drugs higher than 40% for the children when crushed Kaletra® was administered compared to the administration of intact tablets (Best et al., 2011). The observation has provided the rational for this intensive research for the LPV-RTV combination.

Trasi et al. explored in detail the physicochemical API-API interaction and pointed out the influence on the respective dissolution performance, especially regarding the achieved supersaturation. They concluded that the co-formulation of RTV and LPV significantly reduced the amorphous solubility of each drug due to their mixing tendency in the drug rich phase. This mixing seems to stabilize the drug rich phase, which causes a shift in its balance with the molecularly dispersed API and hence reduced the amorphous solubility in the aqueous phase (Trasi and Taylor, 2015a, Trasi and Taylor, 2015b). In a subsequent study, a formulation approach was developed by wet granulation of both APIs in separated matrices using the polymer PVPVA, which proved to be superior compared to the co-granulation approach, tested in neutral phosphate buffer under non-sink conditions. However, in transfer experiments the supersaturated state of RTV could not be maintained despite the separate granulation, indicating still a direct interaction of the APIs, being also present during dissolution (Trasi et al., 2019).

This current study is part of a larger project dedicated lay out ways to better formulate poorly soluble drugs into child-appropriate dosage forms (Kokott et al., 2023). The combination of RTV and LPV was chosen in part because the high medical need for an appropriate paediatric product is still not fully met. Kaletra® oral solution is often considered critical because it contains 42% ethanol (Best et al., 2011). An oral pellet product has been recently developed but is only approved in few global regions and still suffers from challenges in administration to the paediatric patients (Pasipanodya et al., 2018).

Based on the previous knowledge highlighted, the aim of the current study was to further investigate the interaction of RTV and LPV to improve the dissolution, aiming for the highest achievable and maintained supersaturation. The approach of separating the APIs was continued, however, focusing on the use of different polymers, to predominantly isolate the onsets of dissolution. As second polymer HPMCAS in three different grades was selected. The different degrees of substitution enabled the advantage to selectively modify the dissolution profile, allowing for a fast initial release up to strongly delayed release kinetics (Butreddy, 2022). The great potential of HPMCAS as ASD matrix polymer as well as external stabilizer is well described and could provide an additional benefit (Curatolo et al., 2009; Monschke and Wagner, 2020; Müller et al., 2021). For proper evaluation, the prepared combination approaches were finally tested under non-sink conditions according to an already published transfer model (Müller et al., 2021). Taking into consideration, that single phase dissolution approaches are prone to overestimate supersaturation effects, a biphasic dissolution setup was implemented and modified according to Xu et al. (Xu et al., 2017). Furthermore, the biphasic dissolution test should shed light on the complex interplay of the APIs while dissolution, precipitation and partition into the sink compartment occur simultaneously.

## 2 Materials and methods

### 2.1 Materials

The model APIs for this study were RTV (Desano Pharmaceuticals, China) and LPV (Arene Life Sciences, India). The polymers for the preparation of the ASDs were either Kollidon® VA 64 fine (PVPVA, BASF, Germany) or the three different grades of AQOAT® LMP, MMP and HMP (HPMCAS, Shin Etsu, Japan). The film-coated tablet Kaletra® 200/50 mg was chosen as reference product. Fasted State Simulated Gastric Fluid (FaSSGF) and Fasted State Simulated Intestinal Fluid (FaSSIF) used as biorelevant dissolution media were prepared using ready-to-use powder mixtures (Biorelevant.com, United Kingdom). For each experiment, the media were freshly prepared according to the supplier manual. Decanol (Sigma Aldrich Chemie GmbH, Germany) was utilized as organic phase in the biphasic dissolution setup. Ammonium acetate, methanol HPLC grade and hydrochloric acid (all VWR Chemicals, Germany) were used for the preparation of the mobile phase for HPLC analysis. Polylactide (PLA) (Bavaria Filaments, Germany) was used as material for the additional 3D printed paddle for the biphasic dissolution studies.

### 2.2 Methods

#### 2.2.1 ASD preparation

The ASDs investigated in this study were produced via hot-melt-extrusion (HME) using a co-rotating twin screw extruder (ZSE 12, Leistritz, Germany). An overview about the different produced ASDs is given in Table 1.

**Table 1:** Compositions of formulations prepared via HME.

Formulation code	API	Polymer	Drugload / wt%
20% RTV_PVPVA	RTV	PVPVA	20
30% RTV_PVPVA	RTV	PVPVA	30
40% RTV_PVPVA	RTV	PVPVA	40
20% RTV_HPMCAS 5.5	RTV	HPMCAS LMP (5.5)	20
40% RTV_HPMCAS 5.5	RTV	HPMCAS LMP (5.5)	40
20% LPV_PVPVA	LPV	PVPVA	20
40% LPV_PVPVA	LPV	PVPVA	40
20% LPV_HPMCAS 5.5	LPV	HPMCAS LMP (5.5)	20
40% LPV_HPMCAS 5.5	LPV	HPMCAS LMP (5.5)	40
40% LPV_HPMCAS 6.0	LPV	HPMCAS MMP (6.0)	40
40% LPV_HPMCAS 6.5	LPV	HPMCAS HMP (6.5)	40
20% LPV_5% RTV_PVPVA	LPV & RTV	PVPVA	20 & 5

Before extrusion, API and polymer were blended for 15 min in a turbula mixer (Willy A. Bachofen, Switzerland). For powder feeding a volumetric feeding device (Brabender, Germany) was used at speeds in the range of 40–50% of maximum capacity. A screw configuration with conveying elements and two kneading zones was used at a screw speed of 130 rpm. The extrusion temperature profiles were adjusted individually for the different polymers to achieve a homogenous extrudate. For the PVPVA based ASDs a temperature of 160 °C was selected. For HPMCAS as matrix polymer a higher temperature of 170 °C was needed to overcome the higher melt viscosity. In every setup the extrusion zones were kept at the respective temperatures 160 °C or 170 °C except for the feeding port which was regulated at a lower temperature of 30 °C. Potential degradation was previously excluded by DSC. Extrudates were produced using a 2 mm round die. The extruded filaments were subsequently milled down with a centrifugal mill (Retsch, Germany) using a mesh size of 1 mm and a rotational speed of 12,000 rpm. To facilitate the milling process the filaments were cooled down with dry ice to increase brittleness. The milled ASD powders were classified with a sieve tower (Retsch, Germany). The subsequent studies were performed using the particle sizes listed in Table 2.

**Table 2:** Particle size of the used ASDs, X represents the respective API considered.

Polymer	Particle size fraction in μm
X_PVPVA	500–710
X_HPMCAS	180–500

#### 2.2.2 Particle size distribution

The particle size distribution of the prepared ASDs was investigated in triplicate via laser diffraction (Mastersizer 3000, Malvern, United Kingdom) at an air pressure of 3 bar. For the homogenous transfer of the material to the measurement cell a dry dispersion unit (Malvern, United Kingdom) was used.

#### 2.2.3 Solubility

To determine the equilibrium solubility of RTV and LPV an excess of the respective API powder was dispersed in either 75 ml of FaSSIF or FaSSGF without adding any other excipients. The samples were stirred at 75 rpm for 48 h at 37 ± 2 °C prior to measurement via HPLC. The sampling procedure for solubility was carried out in the same way as described within the dissolution studies in 2.2.7 for the aqueous and in 2.2.8 for the organic phase.

#### 2.2.4 One phase: Small-scale dissolution setup

A small-scale biorelevant dissolution setup was used for fast screening purposes. Screw top glass cylinders with an inner diameter of 32 mm were placed on a stirring plate surrounded by a water bath, to ensure a temperature-controlled environment of 37 °C ± 2 °C. For this purpose, only 75 ml of FaSSIF was used as biorelevant media adjusted to a pH of 6.5. The dissolution behavior of the prepared ASDs was firstly investigated on a small-scale dissolution approach prior to transfer of experiments on pharmacopeial conform dissolution setups. The transferability of the small-scale approach for the use of FaSSIF was proved in a previously conducted study by Mueller et al. (Müller et al., 2021). To exclude potential variations in the dissolution rate triggered by different particle sizes, the used particle size fraction for the respective polymer was kept constant (Table 2.). To keep the dose/volume ratio similar, one tenth of the dose used in the transfer dissolution setup was considered. All dissolutions tests were carried out in triplicate. Sampling procedure as well as the methods for quantification are described in 2.2.7. All dissolution tests were performed using milled ASD powder. For the effect of postprocessing of these ASDS, we refer to our previous publication (Kokott et al., 2023).

#### 2.2.5 One phase: Two-stage transfer dissolution setup

All dissolution studies were conducted at 37 ± 0.5 °C using a Ph. Eur. type 2 apparatus at a rotation speed of 75 rpm. The final volume of the biorelevant media was 750 ml. The single ASDs as well as the ASDs used for the combination approaches were dosed equivalently to the marketed product Kaletra®, meaning 50 mg of RTV and 200 mg of LPV. The two-stage dissolution model was based on the described transfer model of Müller et al. using 250 ml of FaSSGF followed by an equilibration with 500 ml of FaSSIF_conc_ after either 15 min or 120 min of gastric residence at a pH of 1.5 (Müller et al., 2021). All dissolutions tests were carried out in triplicate. Sampling as well as quantification procedure is described in 2.2.7.

#### 2.2.6 Biphasic dissolution testing: The implementation of a sink compartment

To evaluate the potential of the prepared ASDs in a more biorelevant way, selected ASDs were tested in a biphasic dissolution setup. Decanol was added as an “absorption sink compartment” to continuously remove molecularly dissolved API, since the partition process will influence dissolution and precipitation events (Xu et al., 2018). First, the RTV_PVPVA ASD (20%) was tested, to show potential conformity of the model with already gained results in literature before analysing the dissolution-partition profiles for the combination approaches (Denninger et al., 2020; Xu et al., 2017). In several publications octanol is present to generate an additional partition compartment (Deng et al., 2017; Silva et al., 2020; Xu et al., 2017). However, due to major concerns about the user health, the other established organic solvent decanol was preferred in this model. The respective ASDs were first exposed to FaSSGF for 15 min, as previously described in the transfer model. However, in this approach the volumes for FaSSGF (125 ml) and for FaSSIF (250 ml) were halved. Additionally, a rotating basket apparatus was used instead of a paddle and kept lower in terms of rotation speed (60 rpm), to avoid an immediate transition of the particles in the organic phase. Furthermore, an additional paddle was 3D printed from PLA and added to the rotating basket apparatus to ensure sufficient mixing in the organic phase. The subsequent transfer was now performed with a previously saturated (for 1 h) mixture of 250 ml FaSSIF_conc_ and 250 ml decanol (1:1). With the use of a separation funnel 250 ml of the denser phase (FaSSIF_conc_) was first transferred to the vessel followed by a pH adjustment with NaOH to pH of 6.5. Afterwards the organic phase was carefully decanted into the vessel using the separation funnel to generate as little turbulences as possible. Samples were taken from both phases, aqueous and organic, at predefined timepoints. The dissolution tests were performed in duplicate. Sampling procedure as well as the methods for quantification are described in 2.2.7 for the aqueous and in 2.2.8 for the organic phase.

#### 2.2.7 Sampling and quantification in the aqueous phase

Samples from dissolution and solubility studies were collected manually with a 10 ml syringe equipped with a pre filter with a pore size of 1 μm, to ensure a separation of large particles formed during dissolution. Samples with a volume of 10 ml were taken for each timepoint. Via syringe pump 7 ml were discarded over a 0.45 μm PVDF syringe filter (Macherey-Nagel, Germany) to guarantee filter saturation. From the residual sample, 750 μl were transferred to HPLC vials, prefilled with 750 μl methanol and afterwards measured via HPLC. An internal method was used for the determination of LPV and RTV concentrations. An Elite La Chrome system (Hitachi-VWR, Germany) was used. The system was equipped with an L-2200 integrated autosampler, an L-2300 column oven and an L-2400 UV-detector. A 150 × 4.6 mm Nucleosil RP-18 column with a pore size of 5 μm (Machherey-Nagel, Germany) was used. The oven temperature was set to 40 °C and the flow rate was 1 ml/min. The mobile phase was composed of methanol and 5 mM ammonium acetate (pH 4) 65/35, followed by a gradient to 95% methanol to perform an additional wash step for 5 min prior to the return to starting conditions. The chosen detection wavelength was 210 nm. The injection volume was 60 μl.

#### 2.2.8 Sampling and quantification in the organic phase

In the biphasic dissolution approach samples were taken from both phases at predefined timepoints. In the case of the organic phase 3 ml were taken using an Eppendorf-pipette (Eppendorf, Germany) and then diluted 1 to 10 with methanol. A filtration step was omitted here, since it was assumed that the APIs were present in a molecularly dissolved state. For the detection from the organic phase, the previously described method in 2.2.7 was further modified, using the same system and column as described above. The previous composition of methanol and ammonium acetate was changed to 70/30. A final washing step was also implemented in this case. After 13 min the amount of methanol was increased to 95% and maintained for 5 min before returning to the initial composition. The detection wavelength was 210 nm. The injection volume was adapted to 20 μl.

## 3 Results and discussion

### 3.1 One phase small-scale dissolution: Crystalline solubility and formulation screening

The saturation solubility of both APIs is presented in Table 3., with both APIs showing low solubility in intestinal-like conditions. The ionization capacity for RTV in acidic media is well known and could also be detected in this experiment (Law et al., 2004). The difference between the resulting solubility in acidic media compared to neutral environment can be expressed by a change in factor of approx. 40. In contrast, LPV did not show a relevant change in solubility for different pH values.

**Table 3:** Saturation solubility of crystalline RTV and LPV in biorelevant media measured at 37 ± 2 °C; *n* = 3, mean ± s.

Media	RTV solubility	LPV solubility
FaSSGF (pH 1.5)	217.2 ± 11.2 μg/ml	3.2 ± 0.1 μg/ml
FaSSIF (pH 6.5)	5.4 ± 0.6 μg/ml	3.6 ± 0.2 μg/ml

**Fig. 1:**
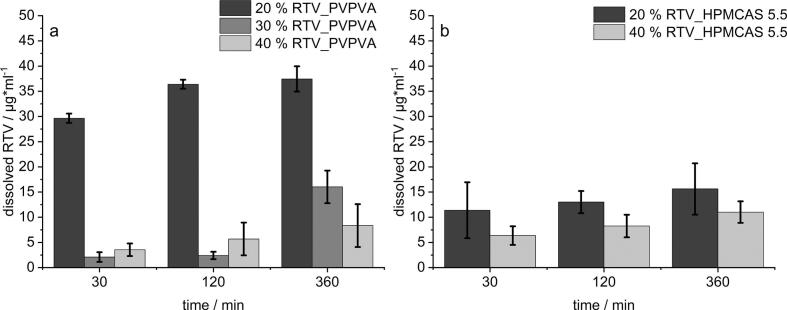
Formulation screening for RTV in a small-scale dissolution setup in 75 ml FaSSIF a) PVPVA, b) HPMCAS; *n* = 3, mean ± s).

After the initial measurement of the equilibrium solubility, the aim of the formulation screening was to find the highest possible drug load and a suitable polymer for both APIs to finally achieve the highest possible supersaturation in FaSSIF maintained for at least 6 h. Therefore, only single ASDs were tested, whereas in the following sections the focus will lay on the investigation of a simultaneous dissolution of both APIs. The results for RTV formulated either with PVPVA in Fig. 1 a) or with HPMCAS 5.5 in b) allow two essential statements. On the one hand, the polymers differed significantly in their performance emphasizing PVPVA as superior alternative for RTV ASDs. The most promising approach with PVPVA (20% RTV) showed a concentration of approx. 40 μg/ml constituting a degree of supersaturation (DS) of 8. In contrast to that the 20% load of RTV in HPMCAS 5.5 did not exceed a concentration of 20 μg/ml. Within this experiment only the HPMCAS grade which dissolves at lowest pH (5.5) was used, since a fast onset of dissolution is typically desired when formulating ASDs. On the other hand, the experiment revealed a limit in drug load for RTV in PVPVA, expressed by a sharp drop in concentration during dissolution when the load was further increased to 30% and 40%, respectively. The amorphous state of the ASDs was previously verified via XRPD and DSC (data not shown).

**Fig. 2:**
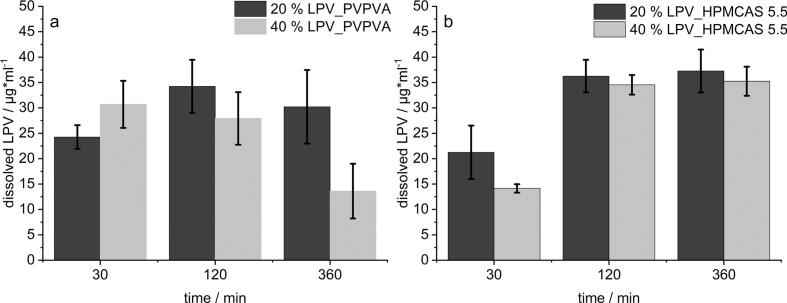
Formulation screening for LPV in a small-scale dissolution setup in 75 ml FaSSIF a) PVPVA, b) HPMCAS; n = 3, mean ± s.

The dissolution results for LPV depicted in Fig. 2 showed a different behavior compared to RTV regarding the suitability of the polymer as well as the drug load. In contrast to RTV, the use of HPMCAS 5.5 was shown to be superior for LPV. This became apparent, especially when looking at the duration the supersaturated solution could be stabilized. The prepared LPV ASDs with PVPVA in Fig. 2 a) revealed for the higher loaded ASDs (40%) a visible decrease in the supersaturation from 30 μg/ml after 120 min to about 10 μg/ml after 360 min. However, the trend was not observed for the 20% loaded ASD. After a slower onset of dissolution, due to the lower dissolution rate of HPMCAS compared to PVPVA, for both drug loads (20% and 40%), sufficiently higher supersaturated solutions (40 μg/ml) could be generated and stabilized for at least 360 min (Fig. 2 b). Interestingly, generation and stabilization of the supersaturation could be achieved independent of the drug load in this case. The drug load only had an influence in the initial phase of dissolution after 30 min.

### 3.2 One phase: Two-stage transfer dissolution approach

#### 3.2.1 Combination approach: The influence of a fast-dissolving polymer

**Fig. 3:**
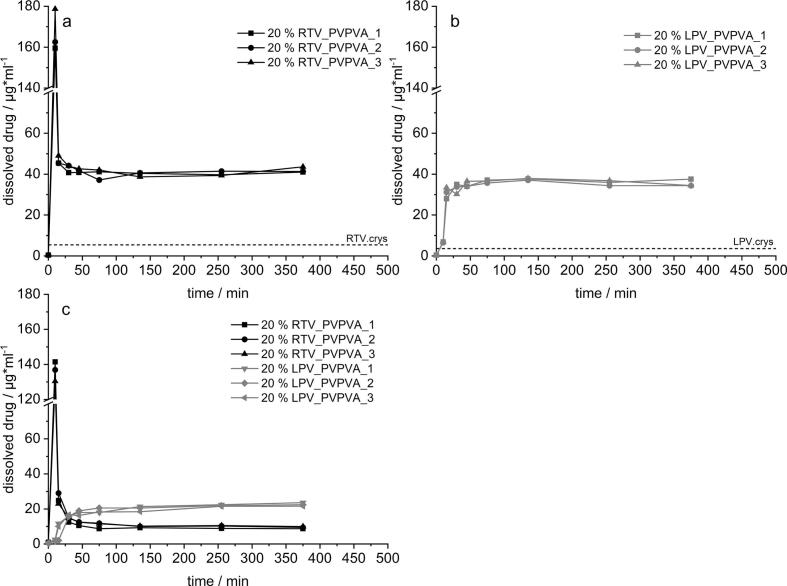
Dissolution profiles in a two-stage transfer dissolution setup a) 20% RTV_PVPVA, b) 20% LPV_PVPVA and c) combination approach of 20% RTV_PVPVA & 20% LPV_PVPVA; *n* = 3.

The most promising formulations obtained from the small-scale experiments were tested for dissolution in combination approaches, starting with the use of only PVPVA as polymer. For visualization of the apparent interaction between the APIs, the dissolution profiles of 20% RTV_PVPVA and 20% LPV_PVPVA are visualised in Fig. 3, both as single dissolution profiles for RTV a) and LPV b) as well as in a combination approach c). When the single RTV ASD was dissolved, the achieved supersaturation after media conversion could be maintained for the observation period with a resulting DS of 8. Despite the absence of a gastric exposure in the small-scale approach, the measured concentration (40 μg/ml) in the two-stage stage dissolution approach were in good agreement. The single dissolution profile of LPV_PVPVA, was also characterized by a sharp increase in concentration within the first 20 min of dissolution ending up in a C_max_ of 35 μg/ml maintained for at least 6 h, while achieving a DS of 10. However, the picture changed dramatically when both ASDs were tested simultaneously in the same vessel. This had an impact on both, the maximum extent of supersaturation as well as the capacity for stabilization, predominantly visible for RTV. After high initial concentrations of RTV in FaSSGF, the concentration dropped to only 10 μg/ml within the first 30 min after the media conversion to FaSSIF which finally resulted in a DS of 2 compared to 8 detected for the single dissolution of RTV. Also, for LPV, the combined dissolution revealed a direct impairment, which is reflected in a halving of the maximum extent of supersaturation to a concentration of 20 μg/ml (DS 5.5).

Notably, these reduced concentrations could not be attributed to crystalline precipitation. According to the findings of Trasi et al., who first described the interaction between both APIs, the formation of an amorphous precipitate in which both APIs are included was hypothesized (Trasi and Taylor, 2015a). The same authors also hypothesized that that the amorphous solubility of each compound is significantly lower in the presence of uncharged molecules which are miscible in the amorphous state due to their lower chemical potential induced by a mixing on a molecular level.

**Fig. 4:**
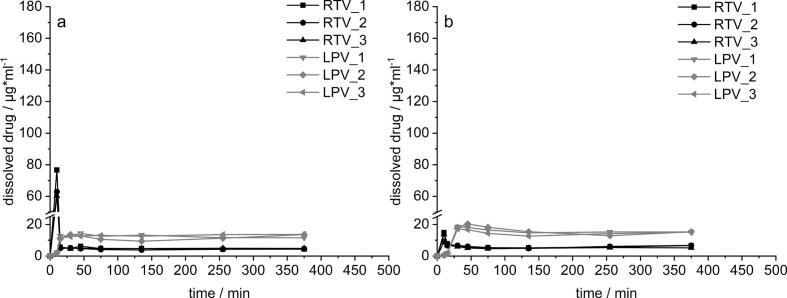
Dissolution profiles in a two-stage transfer dissolution setup a) crushed Kaletra®, b) intact Kaletra®; n = 3.

To get a deeper insight into the high interaction potential of RTV and LPV, the marketed product Kaletra® was also tested for dissolution as depicted in Fig. 4. The intact tablets as well as the crushed tablets were tested to analyze the effect of crushing on the resulting supersaturation of the APIs, since negative effects by this were described in vivo as well as in vitro (Best et al., 2011; Deng et al., 2021). Here, only small differences were detected between the crushed a) and the intact tablet b). The supersaturation of LPV resulting from crushed tablets (15 μg/ml) was lower compared to the intact tablet (20 μg/ml). This result can likely be explained by way more drastic supersaturation reached for RTV in the gastric phase from crushed tablets. Due to the higher specific surface area after crushing, the concentrations of RTV in FaSSGF was approximately 7 times higher than for intact tablets. As a result, the onset of LPV to dissolve was impaired. If the observed differences in LPV concentration are sufficient to explain the different in vivo performance described by Best et al. is, however, questionable (Best et al., 2011). Also, variation of the simulated gastric residence time from here applied 15 min to 120 min did not lead to notable alteration in the dissolution profiles (data not shown).

#### 3.2.2 Combination approach: The influence of a slow-dissolving polymer

**Fig. 5:**
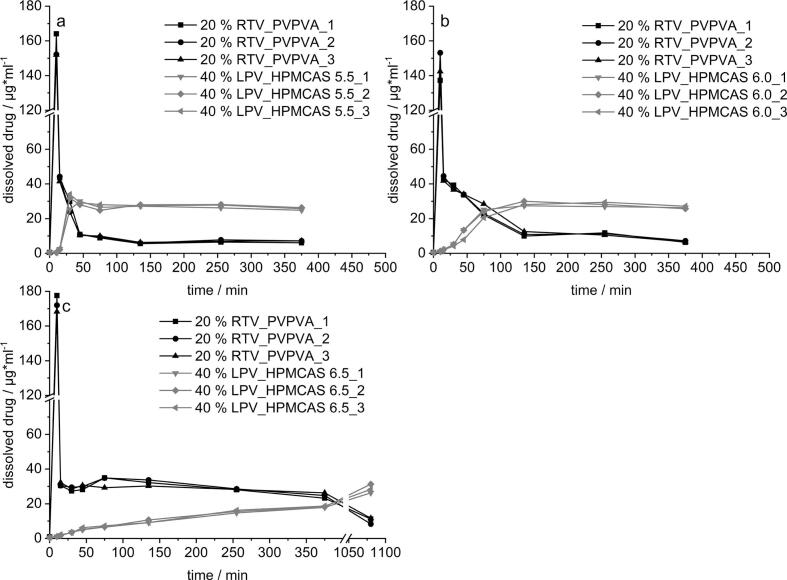
Dissolution profiles in a two-stage transfer dissolution setup for combination approaches a) 20% RTV_PVPVA with 40% LPV_HPMCAS 5.5, b) 20% RTV_PVPVA with 40% LPV_HPMCAS 6.0 and c) 20% RTV_PVPVA with 40% LPV_HPMCAS 6.5; n = 3.

After demonstrating the interaction between RTV and LPV formulated in one polymer matrix (Kaletra®) as well as separately in the same fast-dissolving polymer PVPVA, the slower dissolving HPMCAS was tested as matrix polymer for LPV. The use of HPMCAS as matrix polymer is abundantly discussed in literature, especially its potential to stabilize the supersaturated state during dissolution due to the inhibitory effect on recrystallization (Curatolo et al., 2009; Kawakami, 2017). Based on the results from the small-scale dissolution screening, the 40% LPV load was selected for the combination approaches (Fig. 5). To better explain the effect of a delayed release kinetics of LPV, three different grades of HPMCAS were evaluated, differing in their pH dependent onset of dissolution due to varying substitution ratios of acetyl and succinyl groups (Butreddy, 2022; Tanno et al., 2004).

Each combination was tested with the same ASD for RTV (20% RTV_PVPVA), only the LPV ASD was varied. In Fig. 5 a), the dissolution curves of both APIs are shown, considering an LPV_HPMCAS 5.5 ASD. This polymer offered a dissolution onset at pH 5.5, dissolving immediately after the transfer to FaSSIF, reaching a C_max_ of 30 μg/ml after about 30 min. In comparison to the PVPVA combination seen in Fig. 3 c) (20 μg/ml for LPV), the DS increased from 5 to 8. Despite a stabilization of the supersaturated state of LPV, an abrupt decrease in the RTV concentration was seen directly after the onset of LPV release. However, if the onset of LPV dissolution was delayed for 30 mins with the use of HPMCAS 6.0 (Fig. 5 b) it was observed that the supersaturation of RTV decreased significantly slower and could be maintained for at least 75 min at concentrations of about 30 μg/ml. Interestingly, the prolonged supersaturation of RTV did not affect the C_max_ of LPV. A similar final plateau concentration was achieved as for the formulations with HPMCAS 5.5. This is in line with the assumption that the driving force of decrease in supersaturation is precipitation in a certain molar ratio. Therefore, the slower increase in concentration of LPV directly corresponds to the slower decrease in concentration with the other API RTV. If the onset of dissolution of HPMCAS was further delayed by using HPMCAS 6.5 (Fig. 5 c), the previously observed trend was further extended. The LPV concentration increased extremely slowly, and dissolution was not completed after 6 h. Correspondingly, the RTV concentration decreased equally slowly. At the extended dissolution time of 18 h, the similar plateau concentrations as for the other experiments were reached as could be seen in Fig. 5 c).

The separation in dissolution onset of both APIs was seen as an interesting approach, especially since in vivo the faster releasing one could be rapidly absorbed and thus be removed from the intestinal dissolution medium. Since the separation was only minimally pronounced for HPMCAS 5.5 and the LPV release from HPMCAS 6.5 was likely too slow to be biopharmaceutically reasonable, HPMCAS 6.0 was considered as most relevant and therefore selected for the biphasic dissolution testing.

### 3.3 Biphasic dissolution: The implementation of a sink compartment

**Fig. 6:**
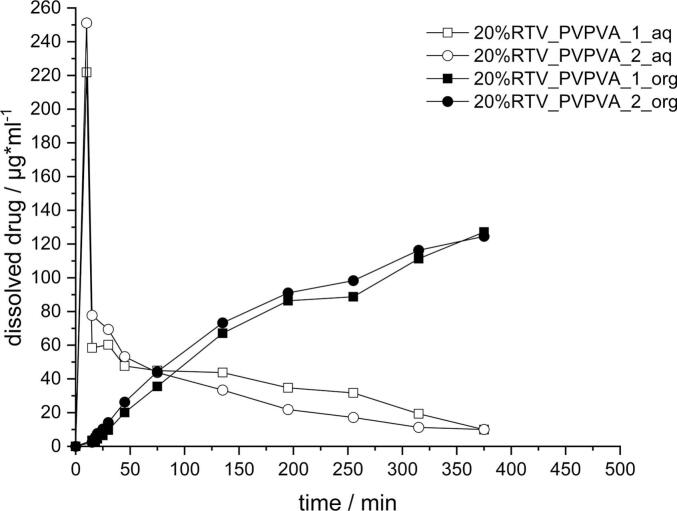
Dissolution-partition profiles in a biphasic dissolution setup for 20% RTV_PVPVA; *n* = 2.

The dissolution-partition profile of the RTV_PVPVA ASD is depicted in Fig. 6, representing the dissolution curves in the aqueous phase with the unfilled symbols whereas, the partition profiles into the organic phase are shown by the respective filled symbols. After the initial rapid increase in FaSSGF (first 15 min), the concentration dropped due to the media conversion. This was followed by a continuous decrease in supersaturation, down to a concentration of 10 μg/ml after 6 h. Parallelly to the decrease of API concentration in the aqueous phase, a continuous partition of the API into the organic phase was observed over a period of 6 h with maximum concentrations of 120 μg/ml. Considering a total dissolved dose of 50 mg, the final mass of RTV detected in decanol was approx.

30 mg, which is comparable to values reported in literature (Denninger et al., 2020). The observed shape of the curve differed to some extend from literature data, which can likely be explained by different protocols for pH adjustments, media selection as well as differences in the apparatus used (Denninger et al., 2020; Xu et al., 2017). Despite these differences, the principal alignment with literature data was seen as indicator that the described setup is suitable to test the proposed hypothesis that separating the dissolution onset for both APIs could be biopharmaceutically advantageous.

**Fig. 7:**
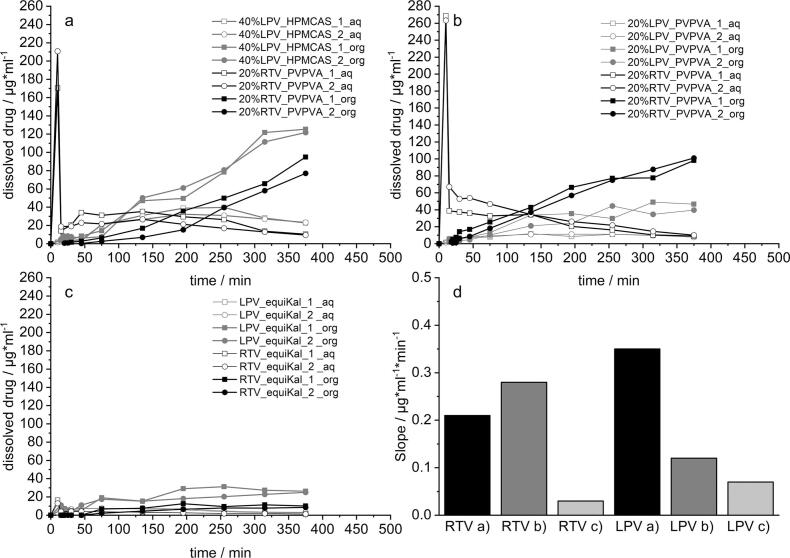
Dissolution-partition profiles in a biphasic dissolution setup for combination approaches a) 20% RTV_PVPVA & 40% LPV_HPMCAS 6.0, b) 20% RTV_PVPVA & 20% LPV_PVPVA, c) ASD equivalent to Kaletra® and d) Slopes of the partition curves for each API calculated from the approaches from a-c); n = 2, mean.

The results from biphasic dissolution testing for three different combination approaches are shown in in Fig. 7. Firstly, the most promising 20% RTV_PVPVA_40% LPV_HPMCAS6.0 a), secondly the approach using PVPVA as polymer for separated ASDs b) and thirdly, an ASD where both APIs are embedded in one PVPVA matrix c). The last one should serve as reference for Kaletra®, which could not be used in this experiment as the high amounts of included surfactants did not allow for separated aqueous and organic phases. The respective slopes of the partition curves of each API for the investigated approaches, calculated by a simple linear regression of the dissolution curves are also depicted d). The slope can be seen as suitable parameter for assessing the different formulation approaches as it describes the rate with which the dissolved APIs partitioned into the organic phase. The increase of RTV in the organic phase was largely similar for both approaches with separated ASDs (a and b), with slopes of 0.22 and 0.28 μg*ml^−1^*min^−1^. Some differences were observed in the very early phase, where the RTV ASD dissolved in presence of LPV_HPMCAS seemed to display a more pronounced lag time. RTV concentrations in the organic phase for both systems steadily increased to maximum concentrations of about 80 μg/ml and 90 μg/ml, which represents for a reduction of approximately 20% compared to the profiles shown for the single analysis of RTV (Fig. 6). More substantial differences were observed for LPV, where the HPMCAS-based ASD led to faster increase. The overall slope was at 0.35 μg*ml^−1^*min^−1^ compared to 0.12 for the PVPVA-based ASD. Also, the finally reached concentrations in the organic phase differed, with values of 120 and 50 μg/ml, respectively.

The most drastic differences were observed when comparing both approaches with the APIs in separated ASDs (a and b) with the combined ASD c). When embedded in one single polymer matrix, the performance for both APIs was substantially decreased. This finding was characterized on the one hand by a lack of supersaturation in the aqueous phase, as well as by very low transition into the decanol phase with maximum concentrations of 10 μg/ml and 25 μg/ml for RTV and LPV, respectively. The results of the biphasic dissolution setup indicate that separating the two studied model APIs in two different ASDs seems to be a relevant advantage for the selected model APIs. The results furthermore supported the presented hypothesis that separating API release by use of different matrix polymers could additionally benefit the biopharmaceutical performance.

## 4 Conclusion

A new formulation approach for the FDC combination of RTV and LPV has been demonstrated in this study. It is assumed that it can serve as a model for the combination of poorly soluble drugs of high structural similarity in ASD formulations.

Initial small-scale screening experiments revealed the superiority of HPMCAS as matrix polymer for LPV regarding the highest achievable drug load as well as the extent and maintenance of supersaturation. However, this was not the case for the formulation with RTV, where the polymer PVPVA, which is also used in the marketed product Kaletra®, performed significantly better. Of even greater relevance was the evaluation of different combination approaches. Contrary to previously published studies it could not be confirmed that the sole separation of the two APIs into a fast dissolving polymer can reduce the known interaction during dissolution (Trasi et al., 2019). Particularly, the supersaturation of RTV was decreased immensely after the onset of LPV dissolution. Compared to the release of RTV alone only, one quarter of the previously measured concentration was detected, whereas LPV concentration decreased by half.

When using the slower dissolving polymer HPMCAS for embedding LPV, the release profiles of the combinations were significantly improved for both APIs. By delaying the onset of LPV release, RTV supersaturation could be stabilized remarkably longer. However, even in this combination, the negative interaction could be observed. It was demonstrated that by selecting the HPMCAS grade, the onset could be tuned to adjust the RTV performance accordingly. The supersaturation of LPV on the other hand was independent from the HPMCAS grade used and achieved in every case the same C_max_.

It was hence hypothesized that separating the dissolution onset of both APIs could increase the biopharmaceutical performance of the formulation. To further stress this hypothesis in a more biorelevant way, a biphasic dissolution setup was implemented. The experiments revealed on the one hand a superiority of the combination approach with HPMCAS to the only use of PVPVA in separated ASDs expressed by a 3 times higher partition of LPV. In addition, the biphasic experiment also demonstrated that in this comparison the embedding of both APIs in a single matrix led to almost no partition into the organic phase and is therefore likely not suitable.

In conclusion, this study has revealed several new insights in the interplay of RTV and LPV and presented a new formulation approach which offers the possibility to further limit the known interaction of the APIs with the final aim to possibly increase the therapeutic efficacy for the patients. However, it should be emphasized that all presented data were based on in vitro dissolution results. Especially with respect to the pharmacokinetic interaction of the two model APIs, in which RTV is used to boost LPV exposure, it cannot be taken for granted that in vitro results actually translate in vivo. Future research should therefore focus on the generation of in vivo results to further confirm the in vitro shown superiority.

## CRediT authorship contribution statement

**Marcel Kokott:** Writing – review & editing, Writing – original draft, Visualization, Validation, Methodology, Investigation, Formal analysis, Data curation, Conceptualization. **Jörg Breitkreutz:** Writing – review & editing, Supervision. **Raphael Wiedey:** Writing – review & editing, Supervision, Investigation.

## Declaration of competing interest

The authors declare that they have no known competing financial interests or personal relationships that could have appeared to influence the work reported in this paper.

## Data Availability

Data will be made available on request.
